# Changes in healthcare use among individuals who move into public housing: a population-based investigation

**DOI:** 10.1186/s12913-018-3109-7

**Published:** 2018-06-05

**Authors:** Aynslie M. Hinds, Brian Bechtel, Jino Distasio, Leslie L. Roos, Lisa M. Lix

**Affiliations:** 1 Department of Community Health Sciences, University of Manitoba, S113-750 Bannatyne Ave, Winnipeg, Manitoba R3E 0W3 Canada; 2 Cross Ministry and Community Partnership Initiatives Community and Social Services, 3rd floor, 10044-108 Street, Edmonton, Alberta T5J 5E6 Canada; 3 Department of Geography, University of Winnipeg, 515 Portage Avenue, Winnipeg, Manitoba R3B 2E9 Canada

**Keywords:** Public housing, Healthcare use, Health services, Health status, Record linkage, Administrative data

## Abstract

**Background:**

Residence in public housing, a subsidized and managed government program, may affect health and healthcare utilization. We compared healthcare use in the year before individuals moved into public housing with usage during their first year of tenancy. We also described trends in use.

**Methods:**

We used linked population-based administrative data housed in the Population Research Data Repository at the Manitoba Centre for Health Policy. The cohort consisted of individuals who moved into public housing in 2009 and 2010. We counted the number of hospitalizations, general practitioner (GP) visits, specialist visits, emergency department visits, and prescriptions drugs dispensed in the twelve 30-day intervals (i.e., months) immediately preceding and following the public housing move-in date. Generalized linear models with generalized estimating equations tested for a period (pre/post-move-in) by month interaction. Odds ratios (ORs), incident rate ratios (IRRs), and means are reported along with 95% confidence intervals (95% CIs).

**Results:**

The cohort included 1942 individuals; the majority were female (73.4%) who lived in low income areas and received government assistance (68.1%). On average, the cohort had more than four health conditions. Over the 24 30-day intervals, the percentage of the cohort that visited a GP, specialist, and an emergency department ranged between 37.0% and 43.0%, 10.0% and 14.0%, and 6.0% and 10.0%, respectively, while the percentage of the cohort hospitalized ranged from 1.0% to 5.0%. Generally, these percentages were highest in the few months before the move-in date and lowest in the few months after the move-in date. The period by month interaction was statistically significant for hospitalizations, GP visits, and prescription drug use. The average change in the odds, rate, or mean was smaller in the post-move-in period than in the pre-move-in period.

**Conclusions:**

Use of some healthcare services declined after people moved into public housing; however, the decrease was only observed in the first few months and utilization rebounded. Knowledge of healthcare trends before individuals move in are informative for ensuring the appropriate supports are available to new public housing residents. Further study is needed to determine if decreased healthcare utilization following a move is attributable to decreased access.

**Electronic supplementary material:**

The online version of this article (10.1186/s12913-018-3109-7) contains supplementary material, which is available to authorized users.

## Background

Public housing is a form of subsidized housing owned and/or managed by government (municipal, provincial/state, or federal). Public housing tenants pay rent geared to income - - usually 30% of the household income [1]. The intent of public housing is to offer a broad safety net to the economically disadvantaged [2].

Public housing residents tend to be in poorer health compared to the general population, with lower self-reported health [3–5], a higher prevalence of chronic diseases (including diabetes, hypertension, asthma) [3, 6–8], injuries [8], and mental health disorders [8–11]. Public housing residents are more likely to engage in risky health behaviors, including smoking, alcohol and drug use, as well as risky sexual behaviors [3, 4, 12–17], and generally have lower levels of physical activity [5, 18–24]. There is evidence, however, that residents’ poor health precedes their application for public housing [25].

Research about healthcare use among public housing residents is limited and inconsistent. McNeill et al. (2009) found that 87% of their sample of 1554 individuals residing in 12 public housing sites in Massachusetts reported having access to a regular health care provider [26]. In Black et al.’s two studies [27, 28], 90% of the older adult participants had received medical care in the past six months, averaging six medical visits during this time, but only 43% of residents had obtained care from a private physician (the rest obtained care from a clinic or hospital-based provider). After adjusting for demographic, health, hospital, and neighbourhood characteristics, children in public housing that had not been redeveloped were significantly more likely to have recurrent use of acute care services when compared with children not residing in public housing or in public housing that had been renovated and redeveloped as part of the Housing Opportunities for People Everywhere (HOPE VI) program [29].

We endeavored to determine if healthcare use changes over the short-term among new residents of public housing. Our research objective was to examine trends in healthcare use one year before and one year after individuals move into public housing. We tested for a change in healthcare use between the two periods. We hypothesized that all forms of healthcare use would increase before the move-in date since health may be related to applying. In a previous study, we found that people who applied to public housing were more likely to have health conditions and were higher users of healthcare services than individuals who were similar in terms of their socioeconomic characteristics [25]. We also hypothesized that all forms of healthcare use would decrease after the move-in date. The reasons for this decrease are potentially multifaceted and could include reduced access to healthcare, an adjustment period after moving in, or to a better housing and financial situation, which may improve health, at least temporarily. We suspected that healthcare use may not remain at a reduced level because public housing residents often have chronic conditions which require ongoing care.

## Methods

### Study cohort

Manitoba is an ethnically diverse Canadian province with a population of approximately 1.3 million, with 55% residing in the City of Winnipeg, the capital. The cohort included all adults (18+ years) who moved into public housing provided by the provincial ministry, Manitoba Housing, between January 1, 2009 and December 31, 2010 and were listed as the primary applicant. There are approximately 35,000 social housing units in Manitoba; of which approximately 13,000 are public housing units spread throughout the province that are directly managed by Manitoba Housing [30]. More than 30,000 individuals reside in public housing units in a year, with approximately half under the age of 20 years [30].

The cohort included individuals registered with the Manitoba Health Services Insurance Plan in the year prior to and in the year following their public housing move-in date. Only new applicants were included; individuals residing in public housing within two years of their 2009/2010 move-in date were excluded. Manitoba Housing requires that people reapply if they wish to switch to a different housing unit; therefore, these people do not have a year out of public housing before their 2009/2010 move-in date. In Churchill, a remote northern Manitoba community, public housing is used to supplement the shortage of affordable market housing. Residents of that community were excluded as it is not possible to distinguish between those paying market rate rents and those living in subsidized units [30]. Individuals living in public housing for less than one year were also excluded.

### Data source

The Population Research Data Repository housed at the Manitoba Centre for Health Policy is a rich collection of anonymized health and social administrative databases linkable at the individual level via a unique scrambled personal health identification number. Previous researchers have identified all residents who applied to and/or who moved into public housing and linked this information to a comprehensive set of health and socioeconomic indicators and outcomes [8, 25, 31].

The Tenant Management System (TMS) was used to indicate residents of Manitoba Housing’s rental housing. The TMS contains information on public housing managed by the provincial government (approximately 2300 buildings and 13,000 units). The number of public housing units has remained fairly constant over time; approximately 59% of the units are located in the Winnipeg health region [30]. Demographic (e.g., sex, birth date, six-digit postal code) and health coverage information (e.g., start and end of coverage dates) was obtained from the Population Registry. The Registry contains this information for all Manitoba residents registered with the provincial health plan (excludes military personnel, the Royal Canadian Mounted Police, and new residents) and is updated every six months (June and December). The Social Assistance Management Information Network provides information on households receiving financial support under the provincial Employment and Income Assistance program. Average household income information from the 2006 Canadian Census was used to create an area-level measure of income (i.e., income quintile).

Information on discharges from all acute and chronic care facilities was obtained from the hospital discharge abstracts database. Up to 25 diagnosis codes are recorded using the International Classification of Diseases, 10th revision, Canadian version (i.e., ICD-10-CA). The physician billing claims database captures all fee-for-service physician visits, which comprises the vast majority of visits. A study from 2004 estimated that 93% of physicians in Winnipeg are remunerated on a fee-for-service basis [32], while two recent studies estimated that 84.1% and 86.4% of patients diagnosed with diabetes in Manitoba were treated by a fee-for-service physician [33, 34]. The diagnosis deemed most responsible for the physician visit is recorded using a three-digit International Classification of Diseases, 9th revision, Clinical Modification (i.e., ICD-9-CM) code. Information about visits to adult emergency departments (EDs) in Winnipeg was obtained from the Emergency Department Information System (EDIS). The EDIS database contains information on urgency of need for treatment, arrival and discharge status, timing of healthcare events in the ED (e.g., registration, triage, initiation of treatment), chief patient complaints, and diagnostic and blood tests [35]. ICD codes are not recorded in the EDIS database and there is no corresponding data available on ED visits outside of Winnipeg. Information about prescription drugs dispensed from community pharmacies, including the dispensation date, drug identification number, and dosage, was obtained from the Drug Program Information Network (DPIN) database. The DPIN database includes some over-the-counter medications. A more detailed description of all of the databases is available on the Manitoba Centre for Health Policy website (http://umanitoba.ca/faculties/health_sciences/medicine/units/chs/departmental_units/mchp/).

### Study variables

Demographic variables included sex and age group (18–24, 25–39, 40–64, 65+ years) and were defined at the move-in date. Geographic, residential mobility, and economic variables were defined for the 365 days prior to the move-in date. Six-digit postal code was used to determine residence before a move to public housing. Region of residence was defined as urban or rural (i.e., Winnipeg or non-Winnipeg). Residential mobility was defined by identifying changes in postal code in the 365 days prior to the move-in date. In Winnipeg, a postal code covers a medium sized apartment or a residential block, while postal code areas are larger outside of the city. Individuals were classified as movers or non-movers depending on whether their postal code changed [36, 37]. The Manitoba Housing move-in and move-out dates were used to determine length of tenancy. Application reason indicated why individuals applied to Manitoba Housing and the move-out reasons were grouped into voluntary moves and eviction.

The economic variables were receipt of income assistance (IA) and income quintile (IQ). Individuals were classified as recipients of IA if they or a member of their household received any form of IA in the 365 days prior to the move-in date [38]. IA is based on financial need as well as other eligibility criteria. IQ is an area-level measure of income based on the average household income in the dissemination area (DA), the smallest geographic level for which Census data are reported [39]. The DAs are sorted from poorest to wealthiest and grouped into quintiles. Each quintile represents approximately 20% of the population. Different household income cut-offs define quintiles for urban and rural areas.

Health status in the 365 days prior to the move-in date was established using diagnosis codes for selected conditions in the physician billing claims and hospital discharge abstracts (Appendix). These conditions have been used previously to describe the health of public housing applicants and/or residents [8, 25]. Mental health conditions included schizophrenia, affective (mood and anxiety) disorders, and substance abuse disorders. Physical health conditions included respiratory illness (e.g., asthma, chronic obstructive pulmonary disease, bronchitis, emphysema), diabetes, hypertension, cancer, arthritis, and injuries. Health status in the 365 days prior to the move-in date was summarized using Aggregated Diagnostic Groups (ADGs) [40, 41]. ADGs are groups of ICD-9-CM/ICD-10-CA codes that represent diagnoses that are clinically similar and for which the expected or actual use of health care services is similar. The John Hopkins Adjusted Clinical Group® (ACG®) Case-Mix System version 9 clusters the ICD codes into 32 mutually exclusive ADGs. A higher ADG score indicates a greater level of comorbidity.

To examine trends in healthcare use, the number of general practitioner (GP) physician visits and the number of specialist visits were summarized for twelve 30-day intervals (i.e., months) before and after the move-in date; these were determined using the date of service and physician type in the physician billings claims database. The number of inpatient hospitalizations in each 30-day interval was calculated using the admission and discharge dates recorded in the hospital discharge abstracts database. Pregnancy-related hospitalizations were not included. Hospitalizations within 24 hours and transfers between facilities were considered a single hospitalization [42]. Two hospitalization measures were defined. First, hospital stays that spanned more than one 30-day interval were counted in each interval to account for variations in length of stay. For the second measure, a hospital stay was counted only in the interval in which an individual was admitted to hospital. The number of different prescription drugs using the fourth-level of the Anatomical Therapeutic Chemical (ATC) classification system in each 30-day interval was determined from the dispensation date in the DPIN database. The fourth-level ATC code denotes the chemical, therapeutic, or pharmacological subgroup and has been used by other researchers to count the number of different drugs [43]. The number of days on each drug was determined from the days’ supply and was used to determine if use spanned more than one interval. Prescription drugs spanning more than one interval were counted in each interval. The number of ED visits in each 30-day interval was also calculated. ED visits overlapping more than one interval were counted in each interval.

### Statistical analysis

Descriptive statistics, including means, standard deviations, and frequencies were used to characterize the cohort. We used regression models to test for linear trends in the percentages/means of the healthcare measures over time. Generalized linear models with generalized estimating equations (GEEs) tested for changes in healthcare. We adopted an unstructured correlation structure, the least restrictive structure, to account for the within-subject correlation over the 30-day intervals. The quasi-likelihood information criterion (QIC) was used to assess model fit [44]. Unadjusted and adjusted models were fit to the data. The unadjusted models included period (pre- and post- move-in date), month (30-day interval), and the period by month two-way interaction. The adjusted models included the period, month, and the period by month two-way interaction as well as demographic (i.e., sex, age group), geographic (i.e., region of residence), economic (i.e., IQ, receipt of IA), residential mobility, and health status characteristics (i.e., physician-diagnosed mental (schizophrenia, mood disorders, substance abuse disorders) and physical health (i.e., injury, diabetes, respiratory illness, arthritis, cancer, hypertension) conditions and ADGs).

Hospitalizations, specialist visits, and ED visits were modeled as dichotomous variables (i.e., no hospitalization/visit, hospitalization/visit in the 30-day interval). The regression coefficients are presented as odds ratios (ORs) along with their corresponding 95% confidence intervals (95% CIs). A negative binomial distribution, appropriate for counts of relatively rare events that exhibit extra-Poisson variation, was adopted for modeling the number of GP visits. The regression coefficients are presented as incident rate ratios (IRRs) along with their corresponding 95% CIs. The number of prescription drugs was modeled using a normal distribution and the regression coefficients are presented as means. All analyses were conducted using SAS software version 9.4 (SAS Institute Inc., Cary NC, USA).

## Results

**Fig. 1:**
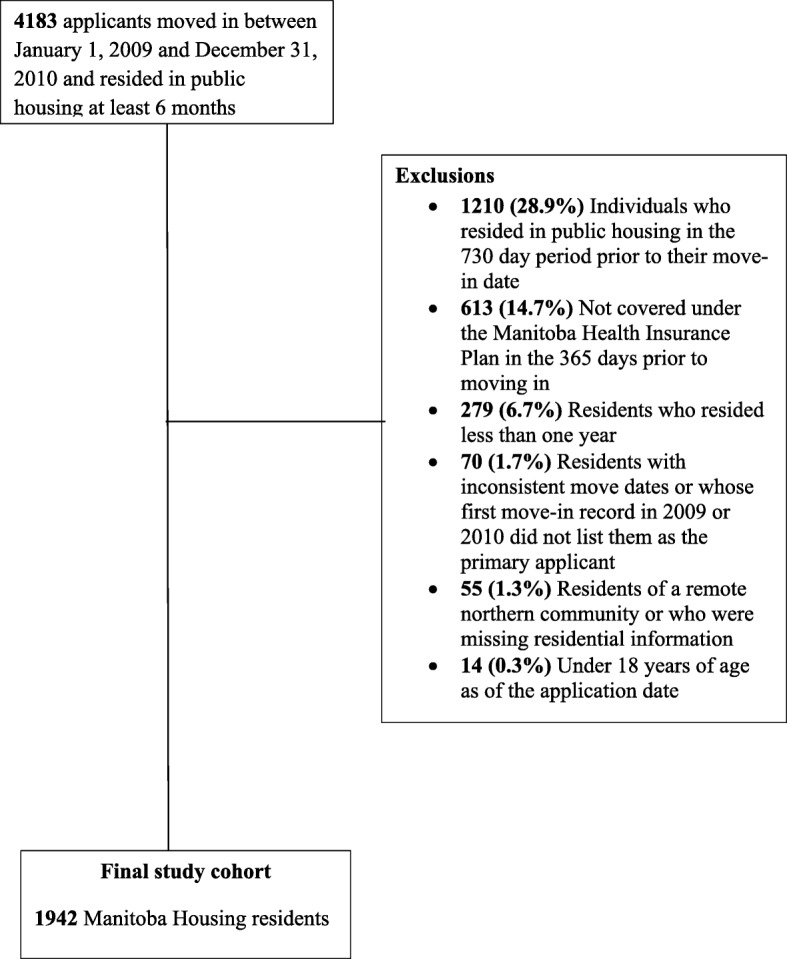
Flow chart for construction of the study cohort

After applying the exclusions, the cohort comprised 1942 (46.4%) of the 4183 adult primary applicants to Manitoba’s rental housing who moved in between January 1, 2009 and December 21, 2010 (Fig. 1). The largest exclusion (28.9%) was because individuals had resided in public housing in the two years before their 2009/2010 move-in date, the majority of whom were continuous residents (i.e., reapplied to move to a different unit) or had moved out and then moved back after a short period of time (i.e., less than one year). There were less than six individuals with multiple housing records who had more than a one year break from residing in public housing before their 2009/2010 move-in date.

### Cohort sociodemographic, health, and housing characteristics

The sociodemographic and health characteristics of the cohort are reported in Table 1. Almost three-quarters of the cohort were female and the average age at the move-in date was 41.7 years (SD = 18.6). The majority were urban residents (54.5%). An IQ gradient showed that as neighbourhood income increased, the percentage of the cohort residing in those areas decreased. More than two-thirds (68.2%) of households received IA. Approximately one-third of the cohort reported an address change in the year prior to the move-in date. The most common physical and mental health conditions were arthritis (26.3%) and affective disorders (32.9%), respectively. The average number of ADGs was 4.52 (SD = 3.14).

**Table 1:** Sociodemographic and health characteristics of the cohort in the 365 days prior to the public housing move-in date (*N* = 1942)

Variables	Categories	*N*	%
Sex	Males	517	26.6
	Females	1425	73.4
Age (years) at Move-in Date	18–24	385	19.8
	25–39	678	34.9
	40–64	621	32.0
	65+	258	13.3
Region	Winnipeg	1058	54.5
	Non-Winnipeg	884	45.5
Income Quintile	Q1 (poorest)	832	42.8
	Q2	478	24.6
	Q3	319	16.4
	Q4	197	10.1
	Q5 (affluent)	93	4.8
	NF^a^	23	1.2
Income Assistance	Yes	1323	68.1
	No	619	31.9
Change in Postal Code	Yes	657	33.8
	No	1285	66.2
Physical Disorders	Arthritis	459	26.3
	Injury	425	21.9
	Respiratory Disease	355	18.3
	Hypertension	286	14.7
	Diabetes	176	9.1
	Ischemic Heart Disease	37	1.9
	Cancer	36	1.9
	Inflammatory Bowel Disease	11	0.6
Mental Disorders	Affective Disorders	638	32.9
	Substance Abuse Disorders	145	7.5
	Schizophrenia	61	3.1

Note. ^a^*NF* Not Found

The housing-related characteristics of the cohort are reported in Table 2. Health/medical reasons were the fourth most common reasons for applying to public housing. During the observation period (January 1, 2009 to March 31, 2013), one-third of the cohort moved out of public housing. Among the movers, 21.9% were evicted and 78.1% moved out voluntarily. The median length of time between the application date and the approval date was 18 days (Q1 = 8, Q3 = 45) and the median length of time between the approval date and the move-in date was 55 days (Q1 = 28, Q3 = 120). In total, the median length of time between the application date and the move-in date was 89 days (Q1 = 47, Q3 = 180).

**Table 2:** Housing-related characteristics of the cohort (*N* = 1942)

Variables	Categories	*N*	%
Application Reason	Overcrowded conditions	632	32.5
	Cannot afford current rent/utilities	516	26.6
	Safety/security	209	10.8
	Health/medical	140	7.2
	Not specified	107	5.5
	To be closer to family/ employment/education	98	5.1
	Family separation	77	4.0
	Physical condition is unsatisfactory	52	2.7
	Notice to vacate	50	2.6
	Unable to maintain current home/yard	33	1.7
	To be closer to medical facilities	28	1.4
Moved-Out Status	Moved out voluntarily	505	26.0
	Evicted	142	7.3
	Did not move out	1295	66.7
Time in Public Housing (days)^a^	Moved	734.1 (256.5)	
	Moved out voluntarily	726.2 (255.0)	
	Evicted	762.5 (260.5)	
	Did not move out	1175.3 (210.9)	

Notes. Application reason was based on information provided prior to the move-in date. Move-out status and time in public housing were based on information obtained after the move-in date (between 2009 and 2013). ^a^Mean (Standard Deviation)

### Healthcare utilization

**Fig. 2:**
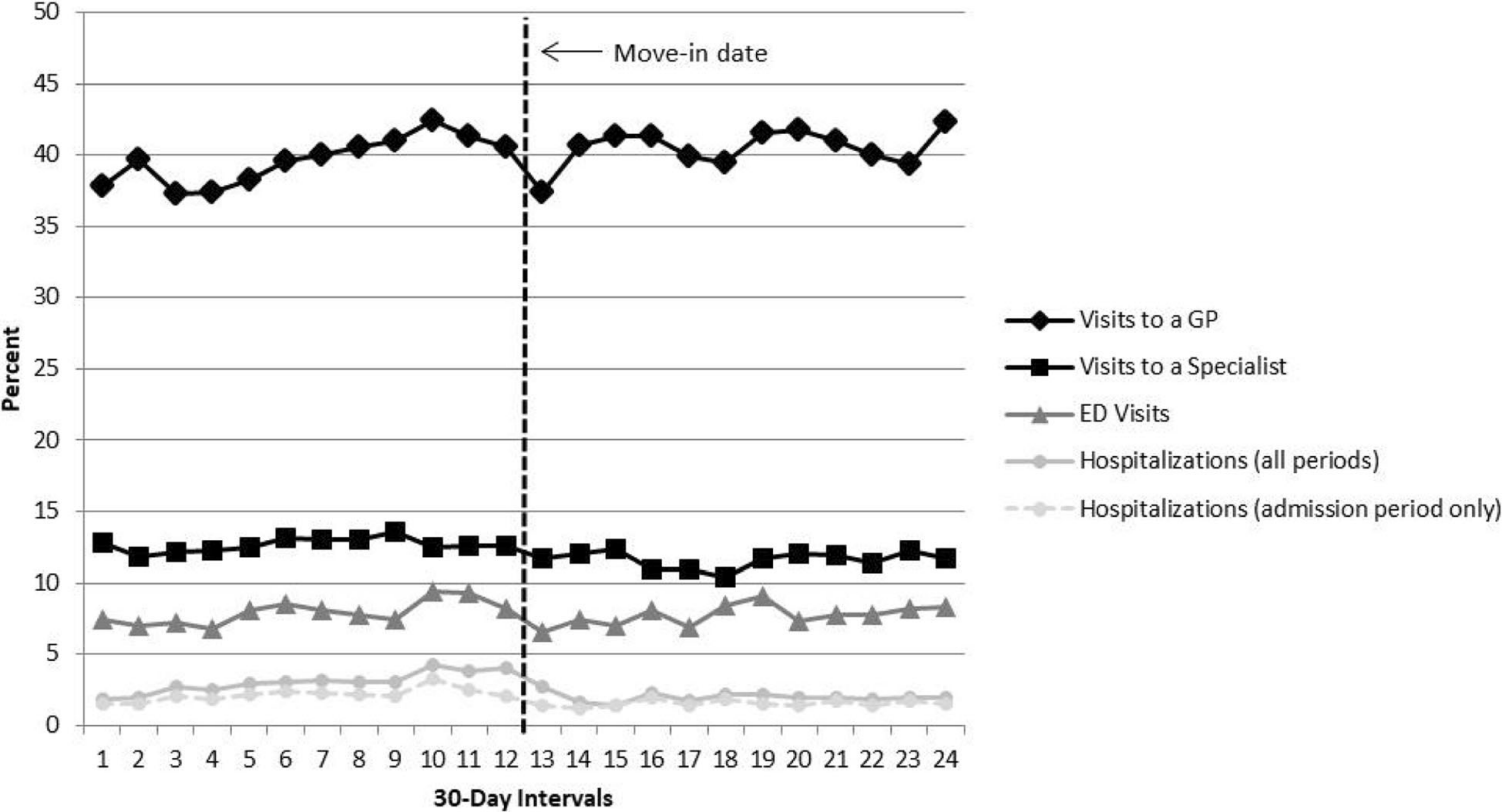
Percentage of the study cohort who were hospitalized, visited an emergency department (Winnipeg residents), and saw a GP or a specialist in the year before and the year after the public housing move-in date. Note. *N* = 1942 for GP visits, specialist visits, and hospitalizations. The N for ED visits (Winnipeg residents) fluctuated between 1067 and 1089 depending on who resided in Winnipeg in any given period

**Fig. 3:**
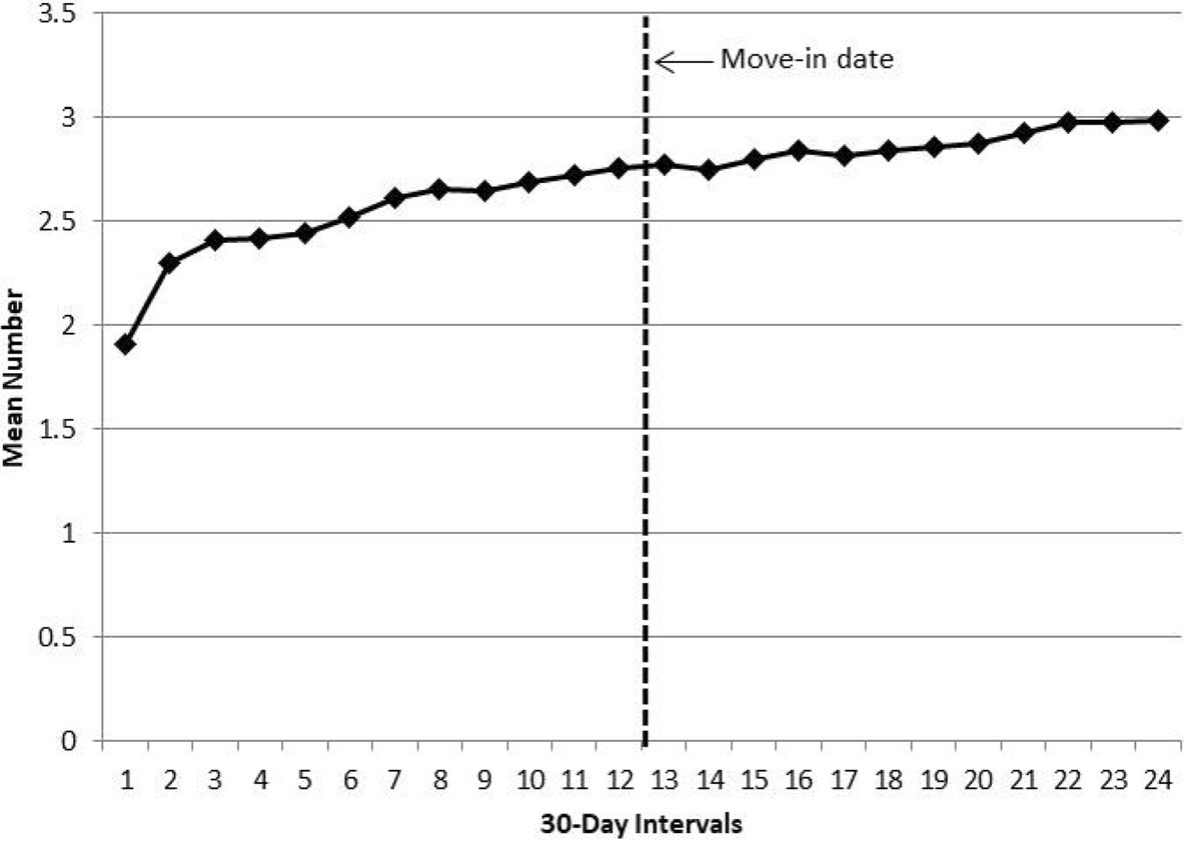
Mean number of prescriptions per 30-day interval filled by the study cohort (*N* = 1942) in the year before and the year after the public housing move-in date

Healthcare use in the twelve 30-day intervals before and after the public housing move-in date is shown in Figs. 2 and 3. Additional file 1: Table S1 shows the percentages/means along with the 95% confidence intervals for the healthcare use measures in each of the 24 30-day intervals. As presented in Fig. 2, in any 30-day interval, between 37.0% and 43.0% of the cohort visited a GP. In the month after the move-in date the percentage of the cohort who visited a GP decreased, but then fluctuated at an increased level comparable to the pre-move-in percentages. There was no evidence that the linear trend in the percentage of GP visits differed in the two periods, *F*(1,20) = 2.8, *p* = 0.11). The cohort averaged 7.42 GP visits (SD = 7.34, median = 6) in the year before and 7.33 GP visits (SD = 6.95, median = 6) in the year after the move-in date. The percentage of the cohort visiting a specialist physician in a 30-day interval ranged between 10.0% and 14.0%; the percentages were lower after the move-in date. However, there was no evidence that the linear trend in the percentage of specialist visits differed in the two periods, *F*(1,20) = 0.3, *p* = 0.56. On average, the cohort had 2.19 specialist physician visits (SD = 4.11, median = 0) in the year before the move-in date and 1.92 specialist physician visits (SD = 3.87, median = 0) in the year after the move-in date.

The percentages of the cohort hospitalized increased before the move-in date, peaking three months before the move-in date at approximately 4.0%, and then decreased after the move-in date and stabilized at around 2.0% at six months after the move-in date. There was evidence that the linear trend in the percentages in the pre-move-in period differed significantly from the linear trend in the post-move-in period, *F*(1, 20) = 31.4, *p* < 0.01. There was a similar pattern in the percentage hospitalized when hospitalizations were only counted in the period in which a person was admitted, except in the two months before the move-in date. The percentage admitted to hospital peaked three months before the move-in date (3.2%) and then decreased, while the percentage hospitalized (accounting for length of stay) remained at an elevated level in the three months before the move-in date. There was no evidence that the linear trend differed in the two periods, *F*(1, 20) = 2.9, *p* = 0.10. In total, 16.9% of the cohort members were hospitalized in the year before the move-in date and 13.9% were hospitalized in the year after.

The percentage of Winnipeg residents who visited an emergency department fluctuated between 7.0% and 10.0% over the 24 30-day intervals. The percentage peaked in the three months prior to the move-in date, declined within the three months after the move-in date, but then the percentages rebounded to the pre-move-in date levels. There was no evidence that the linear trend in the percentage of ED visits differed in the two periods, *F*(1,20) = 0.5, *p* = 0.50. In total, 46.7% and 43.4% of the Winnipeg residents visited an emergency department in the year before and year after the move-in date, respectively. There is no data available on visits to an emergency department outside of Winnipeg.

As shown in Fig. 3, the mean number of different prescriptions filled in a 30-day interval increased over time, from two prescriptions the year before the move-in date to three prescriptions at the end of the first year in public housing. As shown in Additional file 1: Table S1, the 95% CIs for the means at the beginning of the pre-move-in period do not overlap with the 95% CIs for the means at the end of the post-move-in period and there was evidence that the linear trend in the mean number of different prescriptions filled differed in the two periods, *F*(1,20) = 17.1, *p* < 0.01. On average, the cohort filled 6.38 (SD = 5.27, median = 5) different prescriptions in the year before the move-in date and 6.51 (SD = 5.11, median = 6) unique prescriptions in the year after the move-in date.

The estimates and 95% confidence intervals (CIs) for the period by month interactions are presented in Table 3. The period by month interaction was statistically significant for hospitalizations (all intervals) (*p* < 0.01), GP visits (*p* = 0.05), and prescriptions (*p* < 0.01) in the adjusted models. The average change in the odds or rate of utilization was smaller in the post-move-in period than in the pre-move-in period. Additional file 2: Table S2 shows the Chi-square test statistics and *p*-values for the main and interaction effects and Additional file 3: Table S3 shows the estimates and 95% CIs for the variables in each model.

**Table 3:** Unadjusted and adjusted estimates and 95% confidence intervals (CIs) for the period (post-move-in relative to pre-move-in) by month (30-day interval) interaction for healthcare use (*N* = 1942)

Healthcare use	Model	Estimate	95% CI
Hospitalization (OR) (all periods)	Unadjusted	**0.93**	**0.89, 0.97**
	Adjusted	**0.92**	**0.88, 0.96**
Hospitalization (OR) (admission period)	Unadjusted	0.97	0.93, 1.01
	Adjusted	0.97	0.93, 1.02
GP Visits (IRR)	Unadjusted	**0.99**	**0.98, 1.00**
	Adjusted	**0.99**	**0.98, 1.00**
Specialist Visits (OR)	Unadjusted	1.00	1.00, 1.02
	Adjusted	1.00	0.98, 1.02
Prescriptions (mean)	Unadjusted	**−0.03**	**−0.04, −0.02**
	Adjusted	**−0.04**	**−0.05, −0.03**
ED Visits^a^ (OR)	Unadjusted	0.98	0.95, 1.01
	Adjusted	0.98	0.95, 1.10

*Note*. Values in bold-face font are statistically significant at α = 0.05; Covariates in adjusted models = sex, age group, region of residence, income quintile, residential mobility, receipt of IA, physician-diagnosed mental and physical health conditions (i.e., schizophrenia, mood disorders, substance abuse disorders, injury, diabetes, respiratory illness, arthritis, cancer, hypertension), ADGs; ^a^Winnipeg residents only (*N* = 960)

The absolute estimates and 95% CIs for each period are presented in Table 4. The odds of hospitalization in the pre-move-in period changed over time (OR = 1.07; 95% CI 1.04, 1.10), but there was no significant change in the odds in the post-move-in period. Similarly, the odds of an ED visit increased significantly over time in the pre-move-in period (OR = 1.02; 95% CI 1.00, 1.04), but the change in the odds was not statistically significant in the post-move-in period. In the pre-move-in period, the GP visit rate increased slightly (IRR = 1.01, 95% CI 1.01, 1.02), but the change in the GP visit rate was not statistically significant in the post-move-in period. For prescription medications, there was a statistically significant change in both the pre-move-in period (0.06; 95% CI 0.05, 0.07) and post-move-in period (0.02; 95% CI 0.01, 0.03).

**Table 4:** Unadjusted and adjusted estimates and 95% confidence intervals (CIs) for the average rate of change in each period for healthcare use (*N* = 1942)

Healthcare use	Period	Unadjusted		Adjusted	
		Estimate	95% CI	Estimate	95% CI
Hospitalizations (OR) (all periods)	Pre	**1.06**	**1.03, 1.09**	**1.07**	**1.04, 1.10**
	Post	0.98	0.95, 1.01	0.98	0.95, 1.01
Hospitalizations (OR) (admission period only)	Pre	**1.04**	**1.01, 1.07**	**1.04**	**1.01, 1.07**
	Post	1.01	0.98, 1.04	1.01	0.98, 1.05
GP Visits (IRR)	Pre	**1.01**	**1.01, 1.02**	**1.01**	**1.01, 1.02**
	Post	1.00	1.00, 1.01	1.00	1.00, 1.01
Specialist Visits (OR)	Pre	1.00	1.00, 1.02	1.00	0.99, 1.02
	Post	1.00	0.99, 1.01	1.00	0.99, 1.01
Prescriptions (mean)	Pre	**0.05**	**0.04, 0.06**	**0.06**	**0.05, 0.07**
	Post	**0.02**	**0.01, 0.03**	**0.02**	**0.01, 0.03**
ED Visits^a^ (OR)	Pre	**1.02**	**1.01, 1.04**	**1.02**	**1.00, 1.04**
	Post	1.01	0.99, 1.02	1.00	0.99, 1.02

*Note*. Values in bold-face font are statistically significant at α = 0.05; Covariates in adjusted models were sex, age group, region of residence, income quintile, residential mobility, receipt of IA, physician-diagnosed mental and physical health conditions (i.e., schizophrenia, mood disorders, substance abuse disorders, injury, diabetes, respiratory illness, arthritis, cancer, hypertension), ADGs; ^a^Winnipeg residents only (*N* = 960)

## Discussion

This cohort of new public housing residents was primarily comprised of female, urban residents who lived in very low income areas, and received some form of government assistance. Approximately one-third changed their location of residence in the year prior to moving into public housing. This is a high level of residential mobility [36], but is consistent with other studies of this population [25]. On average, the cohort had more than four ADGs, indicating they had a high level of comorbidity, which is consistent with the findings of other researchers [7, 27, 28]. Previous studies reported that public housing residents have a high prevalence of chronic physical health [3] and mental health conditions [7, 9, 10, 13, 45, 46]. The prevalence of respiratory disease (which includes asthma, acute and chronic bronchitis, emphysema, and chronic airway obstruction) in our cohort was high, which may be due to a high prevalence of smoking as reported by other studies [4, 13, 15, 45, 47]. Affective disorders (anxiety and depression) were the most common health conditions. Almost 8% of our cohort had a physician-diagnosed substance abuse disorder; again consistent with other work showing a high prevalence of drug and alcohol use among public housing residents [4, 12–14]. All of these conditions were measured in the year before they moved into public housing. This suggests that public housing in Manitoba accepts and houses individuals with a high burden of disease - individuals who may have trouble obtaining and maintaining employment.

The cohort had a high use of healthcare services both before and after they moved into public housing; however, given the high burden of disease, the amount of healthcare use is not surprising. In any 30-day interval, we found that approximately 40% had a GP visit, 12% had a specialist visits, 8% (of Winnipeg residents) visited an ED, and 2.5% were hospitalized. There was also evidence that healthcare use changed when individuals moved into public housing, but the direction of the change varied by the type of health service. We hypothesized that health may be associated with applying to public housing and hence healthcare use may increase prior to tenancy in public housing, and in fact, 8.7% of the cohort reported a health/medical reason as their motive for applying to public housing. Healthcare use did increase up to approximately three months prior to the move-in date and then within a few months after the move-in date, the percentage of the cohort using all forms of healthcare services decreased, except for those using prescription medications.

Approximately 50% of cohort members applied to public housing three months before their move-in date. Further research is needed to determine whether there is an association between application approval (i.e., approved/not approved, length of time to be approved) and health, and between application reason (i.e., health/non-health) and length of time to be housed.

Prescription use steadily increased over the two-year time period. Specialist visits and hospitalizations were maintained at a level lower in the post-move-in period compared to the pre-move-in date period. The percentage of the cohort who visited GPs and emergency rooms fluctuated over the first year in public housing at levels similar to the year before the move-in date. Our results are consistent with Smith, Alexander, and Easterlow (1997) [48]. They found that healthcare use changed for individuals who moved into medical priority public housing (a practice in Britain of prioritizing individuals with health or mobility problems to receive social housing) [48]. Most people reported their healthcare use decreased; specifically, one in five people visited their family doctor less and had fewer outpatient visits, one in four people had fewer consultant/specialist visits, and one in three people spent less time in hospital.

There was evidence that the average rate of change in the odds/rate of being hospitalized/visiting a GP in the pre-move-in period was higher than in the post-move-in period and that the odds/rate in the pre-move-in period increased over time, while it did not increase in the post-move-in period. Additionally, there was evidence that the average rate of change in the mean use of prescriptions drugs increased over time in both periods, but it increased more quickly in the pre-move-in period. These findings may suggest that public housing interrupted the need for some types of healthcare and that once individuals were housed in public housing they better adhered to their regular source of care.

Wood et al. adopted a similar methodology of using linked administrative data to compare healthcare use before and after people who were homeless moved into public housing; however, they used one year intervals instead of 30-day intervals. They found that access and frequency of some forms of healthcare use in the first year in public housing decreased compared to the year before they moved in [49]. Specifically, there was a significant decrease in access to emergency departments, overnight hospital stays, admissions to the intensive care unit (ICU), receipt of psychiatric care, receipt of mental health services, and use of three prescriptions (i.e., Methadone, Subutex, Suboxone). Also, among the individuals who visited emergency departments, there was little difference in the mean number of visits between the two periods; however, there was a reduction in the mean number of days in hospital, ICU days, days admitted for psychiatric care, mean number of hours of receipt of mental health services, and mean number of prescriptions in the year after the move-in date compared with the year before. Interestingly, the decrease in healthcare use was most pronounced for individuals living in public housing between one and four years. However, when Wood et al. [49] compared the average healthcare use in the three year period before the public housing move-in date with healthcare use in the one year period after the move-in date, some changes in the magnitude of healthcare use were found. Specifically, overnight hospital stays and use of mental health services were more common in the year after the move-in date and the change is the use of psychiatric services varied by program status.

### Study strengths

Our study has a number of strengths. One of the strengths is that we used population-based administrative data. The data is owned and managed by provincial government departments for administrative purposes (e.g., physician reimbursement) and thus are of high quality with no missing information in the main fields. The only ‘missing’ data is the ‘not found’ category for income quintile, which affected 1.2% of the cohort. These data are missing because postal codes were not able to be assigned to a DA or a DA had a small non-institutionalized population.

We linked public housing data to health data at an individual-level to comprehensively examine healthcare before and after the public housing move-in date. Wood et al. compared healthcare use as two periods (pre and post-move-in date) [49]. We divided these periods into twelve 30-day intervals. These shorter units of time allowed us to examine trends, providing evidence there may be health factors precipitating an application to public housing as well as evidence of a transition or adjustment period to public housing.

### Study limitations and future directions

Our cohort was limited to residents of public housing, housing that is directly managed by the province. Approximately 63% of social housing units in the province are not directly managed by the government, but are operated by cooperatives, non-profit groups, and property management agencies. Residents of these forms of social housing were not included as there was no individual-level administrative data available. Also, we excluded individuals who resided in public housing for less than a year and this may have resulted in some selection bias. We suspect that people who have short stays in public housing (i.e., less than one year), are generally less healthy (i.e., more mental health issues and substance use issues) and are more frequent users of healthcare services; therefore, we may have found stronger effects had we included them in the cohort.

Measurement error maybe associated with some of the covariates. For example, the diagnoses of the health conditions are based on physician visits and hospitalizations. Only one diagnosis code is recorded for each physician visit. Consequently, the number of people with any of the health conditions may be underestimated. Residential mobility may have been underestimated if address changes were not reported to the province. However, given the high level of healthcare utilization by the cohort, and that fact that healthcare providers and hospitals require patients to have up-to-date information on their health card, any underestimation in residential mobility is likely minimal.

We did not include a comparison group; consequently we cannot determine whether changes in healthcare use were just reflective of changes in use by the larger population. We plan to conduct a follow-up study using the administrative data to compare healthcare use over time between residents of public housing and a comparison group matched from the general population as well as a group who applied to public housing but did not move-in (i.e., were not approved for public housing or canceled their application to public housing). We were unable to determine what contributed to changes in healthcare use. Potential contributing factors to the decreased use include better access to informal caregivers [48, 50], better housing [51], improved access to social services, including family resource centres [52], more income to spend on nutritious food and recreational activities (income effect) [51], and increased access to other services [53]. A future qualitative research study might shed light on the reasons for changes in use. While the drop in healthcare use may reflect decreased access to health services, this is unlikely as others have found that health services are located close to public housing [53, 54]. Researchers found that residing in a socioeconomically disadvantaged neighbourhood is associated with decreased healthcare access, even after controlling for healthcare supply and individual-level characteristics [55]. A future study is warranted to determine whether this is true in Winnipeg and in Manitoba. The public housing application asks individuals where they want live, but it is not known how closely placement matches preferences. A future study could determine the distance individuals move when they are placed in public housing, describe residential mobility patterns (i.e., are individuals placed in the same neighbourhood as on their application), and determine whether distance to healthcare services (i.e., hospitals, EDs, primary healthcare provider) changes after a move and whether this varies by health region. Additionally, to move within Manitoba Housing, individuals have to reapply; thus, it would is possible to examine residential mobility patterns of public housing residents, and determine how this is related to their pre-public housing location of residence and access to healthcare. Housing administrators would likely find it useful to know where people (with health conditions) are being housed in relation to their healthcare providers and their prior social network. Additionally, since Wood et al. found varying the length of the pre and post move-in periods (one and three years) affected the findings [49], a follow-up study could examine healthcare trends over longer periods. Lastly, further research could examine changes in use by specialist type, changes in physician visits by the reason for the visit (e.g., mental health, physical health, and preventive health (i.e., medical screening) reasons) as well as changes in healthcare use for people with different health conditions.

## Conclusion

In summary, the use of several types of healthcare services (i.e., specialist visits, hospitalizations) declined after people moved into public housing. However, for some forms of healthcare services (GP visits, emergency department visits), the decrease in use was only observed for the first few months after the move-in date; percentages rebounded shortly thereafter. This rebounding effect could be further examined to understand why this occurred.

In general, since public housing residents are high users of healthcare services and tend to experience a high burden of disease, a need exists to strategically locate health and social services in public housing developments, preferably using an integrative, community/client-centred approach, such that there are a range of services in one location (e.g., Community Health Centres or ACCESS Centres) tailored to the community’s needs [52]. As May recommends, housing policy needs to be linked with social policy for service integration [50].

### Additional files

Additional file 1: **Table S1.** Unadjusted Point Estimates and 95% Confidence Intervals (CI) for the Healthcare Utilization Measures over Time. (DOCX 19 kb)

Additional file 2: **Table S2.** Chi-Square Test Statistics and *P*-values for Main and Interaction Effects. (DOCX 17 kb)

Additional file 3: **Table S3.** Model Estimates and 95% Confidence Intervals (CIs). (DOCX 23 kb)

## Appendix

**Table 5:** ICD-9-CM and ICD-10-CA codes for selected health conditions

Condition	ICD-9-CM	ICD-10-CA
Physical		
Respiratory Illness	466, 490–493, 496	J20, J21, J40 – J45
Diabetes	250	E10 – E14
Hypertension	401–405	I10 – I15
Cancer	14–20	C00 – C97
Arthritis	274, 446, 710–721, 725–729, 739	M00 – M03, M05 – M07, M10 – M25, M30 – M36, M65 – M79
Injury	80–99	S00 – S99, T00 – T98
Mental Disorder		
Schizophrenia	295	F20, F21, F25, F232
Affective Disorder	296, 300, 309, 311	F31 – F33, F40 – F42, F44, F48, F99, F341, F380, F381, F410, F411, F412, F413, F418, F419, F431, F432, F438, F450, F451, F452, F530, F680, F930
Substance Abuse Disorder	291, 292, 303–305	F10 – F19, F55

## Abbreviations

ACG: Adjusted Clinical Group
ADG: Aggregated Diagnostic Groups
ATC: Anatomical Therapeutic Chemical
CI: Confidence interval
DA: Dissemination area
DPIN: Drug Program Information Network
ED: Emergency department
GEE: Generalized estimating equations
GP: General practitioner
HOPE: Housing Opportunities for People Everywhere
IA: Income assistance
ICD-10-CA: International Classification of Diseases, 10th revision, Canadian version
ICD-9-CM: International Classification of Diseases, 9th revision, Clinical Modification
ICU: Intensive care unit
IQ: Income quintile
IRR: Incident rate ratio
NF: Not found
OR: Odds ratio
Q1: Quartile 1 (25th percentile)
Q3: Quartile 3 (75th percentile)
QIC: Quasi-likelihood information criterion
SD: Standard deviation
TMS: Tenant Management System
