# Supplementary material for: Changes in healthcare use among individuals who move into public housing: a population-based investigation
Source: BMC Health Serv Res. 2018 Jun 5;18:411. doi: 10.1186/s12913-018-3109-7 (PMC5989341; doi:10.1186/s12913-018-3109-7)
Supplement: Supplementary file 1 — Table S1. Unadjusted Point Estimates and 95% Confidence Intervals (CI) for the Healthcare Utilization Measures over Time. (DOCX 19 kb) [file 12913_2018_3109_MOESM1_ESM.docx]

Additional file 1: Table S1. Unadjusted Point Estimates and 95% Confidence Intervals (CI) for the Healthcare Utilization Measures over Time.

| Time Period | GP Visits | | Specialist Visits | | Emergency Department Visits | | Hospitalizations  (All Periods) | | Hospitalization (Admission Period) | | Prescriptions | |
| --- | --- | --- | --- | --- | --- | --- | --- | --- | --- | --- | --- | --- |
|  | % | 95% CI | % | 95% CI | % | 95% CI | % | 95% CI | % | 95% CI | Mean | 95% CI |
| 1 (12 months prior to move in) | 37.8 | 35.6, 40.0 | 12.7 | 11.3, 14.3 | 7.4 | 5.8, 9.0 | 1.8 | 1.2, 2.4 | 1.5 | 1.0, 2.1 | 1.9 | 1.8, 2.0 |
| 2 | 39.6 | 37.5, 41.8 | 11.8 | 10.4, 13.2 | 7.0 | 5.5, 8.5 | 1.9 | 1.3, 2.5 | 1.5 | 1.0, 2.1 | 2.3 | 2.2, 2.4 |
| 3 | 37.2 | 35.1, 39.4 | 12.1 | 10.7, 13.6 | 7.3 | 5.7, 8.8 | 2.7 | 2.0, 3.5 | 2.0 | 1.4, 2.6 | 2.4 | 2.3, 2.5 |
| 4 | 37.4 | 35.2, 39.5 | 12.3 | 10.8, 13.8 | 6.8 | 5.3, 8.3 | 2.5 | 1.8, 3.2 | 1.9 | 1.3, 2.5 | 2.4 | 2.3, 2.6 |
| 5 | 38.2 | 36.0, 40.4 | 12.5 | 11.0, 14.0 | 8.1 | 6.5, 9.7 | 2.9 | 2.1, 3.6 | 2.2 | 1.5, 2.8 | 2.4 | 2.3, 2.6 |
| 6 | 39.6 | 37.4, 41.8 | 13.2 | 11.7, 14.7 | 8.6 | 6.9, 10.2 | 3.0 | 2.3, 3.8 | 2.4 | 1.7, 3.1 | 2.5 | 2.4, 2.7 |
| 7 | 40.0 | 37.8, 42.2 | 13.0 | 11.5, 14.5 | 8.1 | 6.5, 9.7 | 3.2 | 2.4, 4.0 | 2.2 | 1.6, 2.9 | 2.6 | 2.5, 2.8 |
| 8 | 40.6 | 38.4, 42.8 | 13.0 | 11.5, 14.5 | 7.7 | 6.1, 9.3 | 3.0 | 2.2, 3.7 | 2.1 | 1.5, 2.8 | 2.7 | 2.5, 2.8 |
| 9 | 41.0 | 38.8, 43.2 | 13.5 | 12.0, 15.1 | 7.5 | 5.9, 9.0 | 3.1 | 2.3, 3.9 | 2.1 | 1.4, 2.7 | 2.6 | 2.5, 2.8 |
| 10 | 42.4 | 40.2, 44.6 | 12.5 | 11.0, 14.0 | 9.4 | 7.7, 11.2 | 4.3 | 3.4, 5.2 | 3.2 | 2.5, 4.0 | 2.7 | 2.5, 2.8 |
| 11 | 41.3 | 39.1, 43.5 | 12.6 | 11.1, 14.1 | 9.3 | 7.6, 11.1 | 3.8 | 3.0, 4.7 | 2.5 | 1.8. 3.2 | 2.7 | 2.6, 2.9 |
| 12 | 40.6 | 38.4, 42.8 | 12.6 | 11.1, 14.1 | 8.2 | 6.6, 9.9 | 4.0 | 3.1, 4.9 | 2.1 | 1.4, 2.7 | 2.8 | 2.6, 2.9 |
| 13 | 37.3 | 35.2, 39.5 | 11.7 | 10.3, 13.1 | 6.6 | 5.1, 8.0 | 2.7 | 2.0, 3.5 | 1.4 | 0.9, 1.9 | 2.8 | 2.6, 2.9 |
| 14 | 40.6 | 38.4, 42.8 | 12.0 | 10.6, 13.5 | 7.4 | 5.8, 8.9 | 1.6 | 1.1, 2.2 | 1.1 | 0.6, 1.6 | 2.7 | 2.6, 2.9 |
| 15 | 41.3 | 39.2, 43.5 | 12.4 | 10.9, 13.9 | 6.9 | 5.4, 8.5 | 1.4 | 0.9, 2.0 | 1.3 | 0.7, 1.9 | 2.8 | 2.7, 2.9 |
| 16 | 41.3 | 39.2, 43.5 | 11.0 | 9.6, 12.4 | 8.1 | 6.4, 9.7 | 2.2 | 1.6, 2.9 | 2.0 | 1.3, 2.6 | 2.8 | 2.7, 3.0 |
| 17 | 40.0 | 37.8, 42.1 | 10.9 | 9.5, 12.3 | 6.8 | 5.3, 8.3 | 1.7 | 1.1, 2.3 | 1.3 | 0.8, 1.9 | 2.8 | 2.7, 3.0 |
| 18 | 39.5 | 37.3, 41.7 | 10.4 | 9.0, 11.7 | 8.4 | 6.7, 10.0 | 2.1 | 1.5, 2.8 | 1.8 | 1.2, 2.4 | 2.8 | 2.7, 3.0 |
| 19 | 41.5 | 39.3, 43.7 | 11.7 | 10.3, 13.1 | 9.1 | 7.4, 10.8 | 2.1 | 1.5, 2.8 | 1.5 | 1.0, 2.0 | 2.9 | 2.7, 3.0 |
| 20 | 41.8 | 39.6, 44.0 | 12.0 | 10.6, 13.5 | 7.4 | 5.8, 8.9 | 1.9 | 1.3, 2.5 | 1.4 | 0.7, 1.9 | 2.9 | 2.7, 3.0 |
| 21 | 41.0 | 38.8, 43.2 | 11.9 | 10.5, 13.3 | 7.8 | 6.2, 9.4 | 1.9 | 1.3, 2.5 | 1.8 | 1.2, 2.3 | 2.9 | 2.8, 3.1 |
| 22 | 40.0 | 37.8, 42.2 | 11.3 | 9.9, 12.7 | 7.7 | 6.2, 9.3 | 1.8 | 1.2, 2.3 | 1.4 | 0.9, 2.0 | 3.0 | 2.8, 3.1 |
| 23 | 39.4 | 37.2, 41.6 | 12.3 | 10.8, 13.7 | 8.2 | 6.6, 9.8 | 2.0 | 1.3, 2.6 | 1.7 | 1.1, 2.3 | 3.0 | 2.8, 3.1 |
| 24 (12 months after move in) | 42.3 | 40.0, 44.5 | 11.7 | 10.3, 13.2 | 8.3 | 6.6, 9.9 | 1.9 | 1.3, 2.5 | 1.5 | 1.0, 2.0 | 3.0 | 2.8, 3.1 |
