# Supplementary material for: Changes in healthcare use among individuals who move into public housing: a population-based investigation
Source: BMC Health Serv Res. 2018 Jun 5;18:411. doi: 10.1186/s12913-018-3109-7 (PMC5989341; doi:10.1186/s12913-018-3109-7)
Supplement: Supplementary file 2 — Table S2. Chi-Square Test Statistics and P-values for Main and Interaction Effects. (DOCX 17 kb) [file 12913_2018_3109_MOESM2_ESM.docx]

Additional file 2: Table S2. Chi-Square Test Statistics and P-values for Main and Interaction Effects.

| Variable | GP Visits  (N = 1942) | | Specialist Visits  (N = 1942) | | Emergency Department Visits  (N = 960) | | Hospitalizations  (All Periods)  (N = 1942) | | Hospitalization (Adm. Period)  (N = 1942) | | Prescriptions  (N = 1942) | |
| --- | --- | --- | --- | --- | --- | --- | --- | --- | --- | --- | --- | --- |
|  | χ^2^ | p-value | χ^2^ | p-value | χ^2^ | p-value | χ^2^ | p-value | χ^2^ | p-value | χ^2^ | p-value |
| Period | 2.37 | 0.12 | 1.34 | 0.25 | 0.00 | 0.98 | 0.00 | 0.97 | 1.74 | 0.19 | **159.37** | **< 0.01** |
| Month | **15.15** | **< 0.01** | 0.15 | 0.70 | **4.37** | **0.04** | **4.68** | **0.03** | **5.77** | **0.02** | **181.11** | **< 0.01** |
| Period x Month | **3.86** | **0.05** | 0.02 | 0.89 | 1.58 | 0.21 | **11.90** | **< 0.01** | 1.56 | 0.21 | **40.11** | **< 0.01** |
| Sex | 1.64 | 0.20 | 0.00 | 0.97 | 2.04 | 0.15 | **13.82** | **< 0.01** | **9.12** | **< 0.01** | **4.29** | **0.04** |
| Age Group | 3.83 | 0.28 | **24.73** | **< 0.01** | 3.34 | 0.34 | 5.68 | 0.13 | **5.37** | **0.15** | **125.01** | **< 0.01** |
| Region | 3.24 | 0.07 | **76.47** | **< 0.01** | 0.50 | 0.48 | 3.84 | 0.05 | **11.04** | **< 0.01** | 0.93 | 0.34 |
| Change in  Postal Code | **5.38** | **0.02** | 1.73 | 0.19 | **3.99** | **0.05** | 0.76 | 0.38 | 1.61 | 0.20 | 0.31 | 0.58 |
| Income Quintile | 3.98 | 0.55 | 3.11 | 0.68 | 4.94 | 0.42 | 6.28 | 0.28 | 3.67 | 0.60 | 1.25 | 0.94 |
| Income Assistance | **5.84** | **0.02** | 0.09 | 0.77 | **4.38** | **0.04** | 2.30 | 0.13 | 0.10 | 0.75 | **9.15** | **< 0.01** |
| Schizophrenia | 0.12 | 0.72 | **13.51** | **< 0.01** | 0.70 | 0.40 | **10.24** | **< 0.01** | **9.67** | **< 0.01** | **9.81** | **< 0.01** |
| Affective Disorders | **70.63** | **< 0.01** | **10.18** | **< 0.01** | 0.61 | 0.44 | **4.13** | **0.04** | **4.72** | **0.03** | **25.01** | **< 0.01** |
| Substance Abuse Disorders | **7.93** | **< 0.01** | **12.18** | **< 0.01** | **6.85** | **0.01** | **8.17** | **< 0.01** | **7.70** | **0.01** | 0.28 | 0.60 |
| Injury | 0.67 | 0.41 | **12.52** | **< 0.01** | 0.04 | 0.85 | 0.56 | 0.46 | 1.23 | 0.27 | 0.87 | 0.35 |
| Diabetes | 1.28 | 0.26 | 1.25 | 0.26 | 0.68 | 0.41 | **4.53** | **0.03** | **5.00** | **0.03** | **58.26** | **< 0.01** |
| Cancer | **5.73** | **0.02** | **14.35** | **< 0.01** | 0.03 | 0.86 | 1.88 | 0.17 | 0.92 | 0.34 | 0.00 | 0.97 |
| Respiratory Disease | **14.26** | **< 0.01** | 2.39 | 0.12 | 0.62 | 0.43 | **8.00** | **< 0.01** | 2.36 | 0.12 | **8.24** | **< 0.01** |
| Arthritis | **53.80** | **< 0.01** | 0.61 | 0.43 | 0.01 | 0.93 | **8.14** | **< 0.01** | **7.38** | **0.01** | **25.82** | **< 0.01** |
| Hypertension | **9.84** | **< 0.01** | 0.17 | 0.68 | 2.31 | 0.13 | **11.23** | **< 0.01** | **14.06** | **< 0.01** | **40.85** | **< 0.01** |
| ADGs | **225.95** | **< 0.01** | **155.31** | **< 0.01** | **41.17** | **< 0.01** | **92.24** | **< 0.01** | **103.46** | **< 0.01** | **101.83** | **< 0.01** |

Notes. Values in bold-face font are statistically significant at α = 0.05. ADG = Aggregated Diagnostic Groups.
