# Supplementary material for: Changes in healthcare use among individuals who move into public housing: a population-based investigation
Source: BMC Health Serv Res. 2018 Jun 5;18:411. doi: 10.1186/s12913-018-3109-7 (PMC5989341; doi:10.1186/s12913-018-3109-7)
Supplement: Supplementary file 3 — Table S3. Model Estimates and 95% Confidence Intervals (CIs). (DOCX 23 kb) [file 12913_2018_3109_MOESM3_ESM.docx]

Additional file 3: Table S3. Model Estimates and 95% Confidence Intervals (CIs).

| Variable | Categories | GP Visits  (N = 1942) | | Specialist Visits  (N = 1942) | | Emergency Department Visits  (N = 960) | | Hospitalizations  (All Periods)  (N = 1942) | | Hospitalization (Adm. Period)  (N = 1942) | | Prescriptions  (N = 1942) | |
| --- | --- | --- | --- | --- | --- | --- | --- | --- | --- | --- | --- | --- | --- |
|  |  | IRR | 95% CI | OR | 95% CI | OR | 95% CI | OR | 95% CI | OR | 95% CI | Estimates | 95% CI |
| Period | Post | 1.05 | 0.99, 1.11 | 0.92 | 0.80, 1.06 | 1.00 | 0.83, 1.22 | 1.01 | 0.74, 1.36 | 0.81 | 0.60, 1.10 | **0.62** | **0.53, 0.70** |
|  | Pre | Ref | - | Ref | - | Ref | - | Ref | - | Ref | - | Ref | - |
| Month |  | **1.01** | **1.01, 1.02** | 1.00 | 0.99, 1.02 | **1.02** | **1.00, 1.04** | **1.07** | **1.04, 1.10** | **1.04** | **1.01, 1.07** | **0.06** | **0.05, 0.07** |
| Period x Month | Post | **0.99** | **0.98, 1.00** | 1.00 | 0.98, 1.02 | 0.98 | 0.95, 1.01 | **0.92** | **0.88, 0.96** | 0.97 | 0.93, 1.02 | **-0.04** | **-0.05, -0.03** |
|  | Pre | Ref | - | Ref | - | Ref | - | Ref | - | Ref | - | Ref | - |
| Sex | Male | 0.95 | 0.88, 1.03 | 1.00 | 0.84, 1.18 | 1.19 | 0.95, 1.48 | **1.57** | **1.26, 1.95** | **1.39** | **1.13, 1.70** | **-0.29** | **-0.55, -0.02** |
|  | Female | Ref | - | Ref | - | Ref | - | Ref | - | Ref | - | Ref | - |
| Age Group | 18 – 24 | 1.02 | 0.90, 1.16 | **1.89** | **1.43, 2.50** | 0.98 | 0.59, 1.62 | **0.59** | **0.39, 0.90** | **0.64** | **0.43, 0.95** | **-2.02** | **-2.44, -1.59** |
|  | 25 – 39 | 0.97 | 0.86, 1.10 | **1.32** | **1.01, 1.73** | 0.81 | 0.50, 1.32 | 0.76 | 0.51, 1.11 | 0.75 | 0.52, 1.07 | **-1.77** | **-2.19, -1.35** |
|  | 40 - 64 | 0.94 | 0.84, 1.06 | 1.26 | 0.99, 1.60 | 0.77 | 0.48, 1.24 | 0.82 | 0.58, 1.16 | **0.75** | **0.56, 0.99** | **-0.57** | **-1.00, -0.14** |
|  | 65+ | Ref | - | Ref | - | Ref | - | Ref | - | Ref | - | Ref | - |
| Region | Non-Winnipeg | 1.06 | 1.00, 1.13 | **0.50** | **0.43, 0.58** | 1.39 | 0.63, 3.07 | **1.30** | **1.01, 1.67** | **1.49** | **1.19, 1.86** | 0.09 | -0.10, 0.29 |
|  | Winnipeg | Ref | - | Ref | - | Ref | - | Ref | - | Ref | - | Ref | - |
| Change in  Postal Code | Yes | **1.08** | **1.01, 1.14** | 1.10 | 0.96, 1.25 | **1.22** | **1.01, 1.47** | 1.12 | 0.87, 1.45 | 1.15 | 0.93, 1.43 | -0.05 | -0.24, 0.13 |
|  | No | Ref | - | Ref | - | Ref | - | Ref | - | Ref | - | Ref | - |
| Income Quintile | Q1 (poorest) | 1.06 | 0.93, 1.21 | 0.72 | 0.51, 1.03 | 0.82 | 0.42, 1.61 | 0.72 | 0.47, 1.10 | 0.76 | 0.53, 1.09 | 0.06 | -0.37, 0.49 |
|  | Q2 | 1.03 | 0.90, 1.18 | 0.72 | 0.51, 1.02 | 0.77 | 0.39, 1.53 | 0.72 | 0.47, 1.10 | 0.78 | 0.55, 1.11 | -0.06 | -0.49, 0.38 |
|  | Q3 | 1.04 | 0.91, 1.19 | 0.73 | 0.51, 1.05 | 1.07 | 0.50, 2.26 | **0.64** | **0.41, 0.99** | 0.68 | 0.46, 1.01 | -0.01 | -0.47, 0.45 |
|  | Q4 | 1.02 | 0.88, 1.19 | 0.77 | 0.53, 1.13 | 1.24 | 0.56, 2.74 | 0.73 | 0.45, 1.18 | 0.77 | 0.50, 1.18 | 0.01 | -0.46, 0.49 |
|  | NF | 0.87 | 0.67, 1.14 | 0.65 | 0.31, 1.34 | 0.83 | 0.31, 2.20 | 1.83 | 0.77, 4.31 | 0.96 | 0.52, 1.80 | -0.21 | -1.25, 0.83 |
|  | Q5 (affluent) | Ref | - | Ref | - | Ref | - | Ref | - | Ref | - | Ref | - |
| Income Assistance | Yes | **1.11** | **1.02, 1.20** | 0.97 | 0.81, 1.17 | **1.35** | **1.01, 1.80** | 0.78 | 0.57, 1.05 | 0.96 | 0.77, 1.21 | **0.35** | **0.13, 0.57** |
|  | No | Ref | - | Ref | - | Ref | - | Ref | - | Ref | - | Ref | - |
| Schizophrenia | Yes | 1.03 | 0.87, 1.23 | **2.88** | **1.93, 4.29** | 1.23 | 0.80, 1.88 | **2.91** | **1.87, 4.52** | **1.99** | **1.43, 2.77** | **0.90** | **0.39, 1.40** |
|  | No | Ref | - | Ref | - | Ref | - | Ref | - | Ref | - | Ref | - |
| Affective Disorders | Yes | **1.33** | **1.25, 1.41** | **0.78** | **0.68, 0.91** | 0.92 | 0.75, 1.13 | **0.79** | **0.64, 0.99** | **0.80** | **0.65, 0.98** | **0.56** | **0.35, 0.78** |
|  | No | Ref | - | Ref | - | Ref | - | Ref | - | Ref | - | Ref | - |
| Substance Abuse Disorders | Yes | **1.17** | **1.06, 1.30** | **0.64** | **0.50, 0.83** | **1.57** | **1.17, 2.10** | **1.75** | **1.27, 2.40** | **1.65** | **1.24, 2.20** | 0.11 | -0.29, 0.51 |
|  | No | Ref | - | Ref | - | Ref | - | Ref | - | Ref | - | Ref | - |
| Injury | Yes | 1.03 | 0.96, 1.10 | **0.75** | **0.63, 0.88** | 0.98 | 0.78, 1.23 | 0.91 | 0.71, 1.17 | 0.89 | 0.72, 1.09 | -0.12 | -0.38, 0.13 |
|  | No | Ref | - | Ref | - | Ref | - | Ref | - | Ref | - | Ref | - |
| Diabetes | Yes | 1.06 | 0.96, 1.18 | 1.14 | 0.92, 1.43 | 1.16 | 0.83, 1.62 | **1.44** | **1.06, 1.94** | **1.41** | **1.08, 1.84** | **2.31** | **1.79, 2.83** |
|  | No | Ref | - | Ref | - | Ref | - | Ref | - | Ref | - | Ref | - |
| Cancer | Yes | **0.79** | **0.66, 0.95** | **2.71** | **1.91, 3.86** | 1.08 | 0.50, 2.32 | 0.77 | 0.53, 1.13 | 0.84 | 0.59, 1.20 | 0.02 | -0.90, 0.94 |
|  | No | Ref | - | Ref | - | Ref | - | Ref | - | Ref | - | Ref | - |
| Respiratory Disease | Yes | **1.15** | **1.07, 1.22** | 0.88 | 0.76, 1.03 | 1.10 | 0.88, 1.39 | **0.69** | **0.53, 0.90** | 0.84 | 0.67, 1.05 | **0.39** | **0.13, 0.64** |
|  | No | Ref | - | Ref | - | Ref | - | Ref | - | Ref | - | Ref | - |
| Arthritis | Yes | **1.33** | **1.24, 1.42** | 0.94 | 0.80, 1.10 | 0.99 | 0.78, 1.26 | **0.70** | **0.55, 0.89** | **0.74** | **0.60, 0.92** | **0.74** | **0.46, 1.01** |
|  | No | Ref | - | Ref | - | Ref | - | Ref | - | Ref | - | Ref | - |
| Hypertension | Yes | **1.14** | **1.06, 1.24** | 1.05 | 0.85, 1.29 | 1.30 | 0.96, 0.76 | **1.56** | **1.22, 2.00** | **1.57** | **1.26, 1.33** | **1.40** | **1.00, 1.81** |
|  | No | Ref | - | Ref | - | Ref | - | Ref | - | Ref | - | Ref | - |
| ADGs |  | **1.13** | **1.12, 1.14** | **1.23** | **1.20, 1.26** | **1.14** | **1.10, 1.18** | **1.32** | **1.28, 1.37** | **1.29** | **1.26, 1.33** | **0.28** | **0.23, 0.32** |

Note. Values in bold-face font are statistically significant at α = 0.05. ADG = Aggregated Diagnostic Groups.
